# Supplementary material for: Exploring running styles in the field through cadence and duty factor modulation
Source: PLoS One. 2023 Dec 7;18(12):e0295423. doi: 10.1371/journal.pone.0295423 (PMC10703220; doi:10.1371/journal.pone.0295423)
Supplement: Attachment — Submitted filename: rebuttal letter - 230621.pdf [file pone.0295423.s003.pdf]

Reference number: PONE-D-23-18858 (previous submission PONE-D-23-16206)

Exploring running styles in the field through cadence and duty factor modulation

Dear dr. L. A. Peyré-Tartaruga and editorial office,

Thank you for evaluating our manuscript and for giving us the opportunity to resubmit.

We have made the following changes to the manuscript in response to the concerns of the editorial office:

- We have included the reference numbers of both the original ethical application (VCWE-2019–006R1) and the amendment (VCWE-2021-043) in the method section of the manuscript.
- We have included the original ethical application (VCWE-2019–006R1) in Dutch, with the English translations in comments in the pdf file.
- We have included the amendment (VCWE-2021-043) in Dutch, with the English translations in comments in the pdf file.
- We have included the approval emails from our IRB for both the original ethical application (VCWE-2019–006R1) and the amendment (VCWE-2021-043).
- We have included the informed consent form for this study in English.
- We have included the participant information form for this study in English.

We believe that by making those changes and including the additional files we have addressed the concerns of the editorial office. Please find below the message from the editorial office.

Thank you for your time and consideration.

Sincerely,

Anouk Nijs, Msc.	Dr. Melvyn Roerdink	Prof. Dr. Peter J. Beek
a.nijs@vu.nl	m.roerdink@vu.nl	p.j.beek@vu.nl

Department of Human Movement Sciences, Faculty of Behavioural and Movement Sciences,
Vrije Universiteit Amsterdam, The Netherlands

PONE-D-23-16206
Exploring running styles in the field through cadence and duty factor modulation PLOS ONE

Dear Dr. Nijs,

I am writing to you about your appeal on the editorial decision for your submission to PLOS ONE above.

After careful consideration of the manuscript, the reasons for the previous rejection, and your reasons for appealing, we are upholding the decision to reject the manuscript.

As you are aware, manuscripts submitted to PLOS ONE are assessed based on the journal's publication criteria. We have concerns on the contents of the manuscript including that the approval document provided did not match the study presented in the manuscript. Furthermore, the approval number on the manuscript (VCWE-2019–006R1) did not match the one provided in the email from your IRB (VCWE-2021-043)

Considering those concerns, the manuscript does not currently meet our criteria for publication requiring that the research meets all applicable standards for the ethics of experimentation and research integrity.

However, if you are able to provide a copy of the original approval document issued by your IRB (i.e. VCWE-2019–006R1) and an English translation, as well as the documents that you have mentioned in your appeal email, we do feel that a revised manuscript may be suitable for consideration. This would however need to be considered as a new submission.

If you are able to revise the manuscript as indicated above and submit a new manuscript to PLOS ONE, please refer to the original submission in the cover letter.

Thank you for your interest in PLOS ONE.

Best wishes,

Anushmathi PM
Editorial Office
PLOS ONE
